# PHyPO: Priority-based Hybrid task Partitioning and Offloading in mobile computing using automated machine learning

**DOI:** 10.1371/journal.pone.0314198

**Published:** 2024-12-12

**Authors:** Shehr Bano, Ghulam Abbas, Muhammad Bilal, Ziaul Haq Abbas, Zaiwar Ali, Muhammad Waqas

**Affiliations:** 1 School of Computing and Engineering, University of Huddersfield, Huddersfield, United Kingdom; 2 Telecommunications and Networking (TeleCoN) Research Center, GIK Institute of Engineering Sciences and Technology, Topi, Pakistan; 3 Faculty of Computer Science and Engineering, GIK Institute of Engineering Sciences and Technology, Topi, Pakistan; 4 School of Computing and Communications, Lancaster University, Lancaster, United Kingdom; 5 Faculty of Electrical Engineering, GIK Institute of Engineering Sciences and Technology, Topi, Pakistan; 6 School of Computing and Mathematical Sciences, Faculty of Engineering and Science, University of Greenwich, London, United Kingdom; 7 School of Engineering, Edith Cowan University, Perth, WA, Australia; Sejong University, KOREA, REPUBLIC OF

## Abstract

With the increasing demand for mobile computing, the requirement for intelligent resource management has also increased. Cloud computing lessens the energy consumption of user equipment, but it increases the latency of the system. Whereas edge computing reduces the latency along with the energy consumption, it has limited resources and cannot process bigger tasks. To resolve these issues, a Priority-based Hybrid task Partitioning and Offloading (PHyPO) scheme is introduced in this paper, which prioritizes the tasks with high time sensitivity and offloads them intelligently. It also calculates the optimal number of partitions a task can be divided into. The utility of resources is maximized along with increasing the processing capability of the model by using a hybrid architecture, consisting of mobile devices, edge servers, and cloud servers. Automated machine learning is used to identify the optimal classification models, along with tuning their hyper-parameters, which results in adaptive boosting ensemble learning-based models to reduce the time complexity of the system to *O*(1). The results of the proposed algorithm show a significant improvement over benchmark techniques along with achieving an accuracy of 96.1% for the optimal partitioning model and 94.3% for the optimal offloading model, with both the results being achieved in significantly less or equal time as compared to the benchmark techniques.

## Introduction

### Motivation

The demand for Mobile Computing (MC) is rising due to the portability of User Equipment (UE). MC has a large number of applications due to the successful embedding of more resources on smaller chips, and computational offloading is one of them. Nowadays, a UE has enough processing capabilities to support online gaming [1], and applications based on Augmented Reality (AR), Virtual Reality (VR) [2], big data analysis, healthcare, automation, smart control, and video analysis [3–6]. The other applications include collaborative computing, memory replication, content delivery, and web content optimization [7]. Nowadays, due to the heavy software and run-time interaction with other players, most of the games are played on the cloud server. These games require a high-speed update in the game logic and score, which is attained via MEC [1, 8]. In healthcare, the 3-tier architecture is used by medical advisors to attend to and treat patients anywhere in the world in the least amount of time [9]. Another application of MC in healthcare is the usage of UE sensors like gyroscopes and accelerometers to detect the fall of a person in case of a stroke or any other accident and generate an alert message [10]. Big data analysis in the Internet of Things (IoT) also requires high bandwidth and other architecture to deliver the data to the cloud using Mobile Cloud Computing (MCC). As the UEs are situated near the IoT devices, they are used to deliver the data to the cloud server and then deliver the actions accordingly to the receiving nodes [11]. MC is used in Vehicle-to-Vehicle (V2V) communication for the same purposes as the usage of UEs to deliver the data to edge nodes eradicating the need to install new resources [12]. The usage of mobile applications based on AR and VR has also increased in the recent era. The redundancy in AR and VR application data creates a bottleneck in the back-haul link, which is reduced by intelligent caching introduced in the system by Mobile Edge Computing (MEC) [2]. MEC and MCC are also used in AR and VR to reduce the latency and energy consumption of UE [13]. To mitigate the risks to IoT device data in case of cyber-attacks (e.g. spoofing, DDoS, MITM, etc.), the data is analyzed using different applications deployed in UEs, or edge/cloud servers [14]. In case of a threat or attack, hardware or software-based solutions can be implemented in a short amount of time. The high processing also costs the UE in the form of latency and more battery consumption [4].

The high processing capability in MC costs the UE in the form of latency and higher battery consumption [4]. To overcome these issues, researchers introduced a model of Mobile Cloud Computing (MCC) [15], in which the tasks are offloaded and processed on the cloud server. Due to the high latency introduced in the system because of the distance between UE and the cloud server [16], researchers introduced partial offloading in MCC [17], in which a task is divided into a fixed number of sub-tasks, and is then partially offloaded to the cloud server. To further decrease the latency issues, researchers shifted towards Mobile Edge Computing (MEC) [18], which comprises an edge server nearer to the UE as compared to the cloud server. This model eliminates the latency issue, but it has resource limitations, as compared to the cloud. The service rate of MEC is improved by the introduction of partial offloading, with the number of partitions of the task being fixed [19]. The technique of partial offloading in MEC has reduced the latency and energy consumption of the UE, along with an increase in the service rate [20]. The rapid growth in production and demand for the aforementioned applications require more processing hardware, which cannot yet be integrated on a smaller scale. The other solution is the intelligent usage of existing resources, which is explored in this research.

### Problem definition

The processing capability of a UE is constrained by its battery, memory, and Central Processing Unit (CPU). In the case of MCC, the resources are unlimited, but the latency introduced in the system is unsolicited. The battery consumption of a UE is also high in the case of MCC [16]. MEC reduces the energy consumption along with the latency of processing but has a limited amount of resources because of which the service rate of MEC drops in high traffic cases. In the case of a time-sensitive task, higher traffic in the network can cause a bottleneck. The usage of Machine Learning (ML) can help solve these issues, but the selection of an optimal algorithm and parameters is quite difficult by manual testing.

The identification of the optimal number of partitions and calculation of the offloading destination for them, with the least energy and time consumption, can take a lot of time if done using traditional mathematical calculations. The usage of ML algorithms can help solve this issue in time complexity of *O*(1) but the selection of an optimal model and parameters is quite difficult by manual testing. Due to a huge number of ML algorithms, it is nearly impossible to test the data on each one, with each possible combination of parameters.

### Contributions

**Fig 1:**
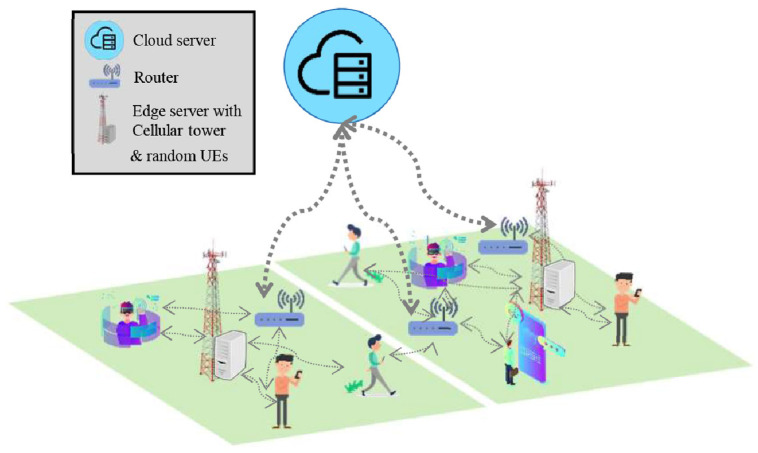
Overview of mobile-edge-cloud server-based system.

This research introduces an algorithm, called Priority-based Hybrid task Partitioning and Offloading (PHyPO), which introduces a hybrid mobile-edge-cloud server-based system where the architecture is 3-tier, which uses a router for cloud server communication, as shown in Fig 1. The figure presents the network scenario considered in this research, in which there are several edge servers and a single cloud server, which are used for partial offloading by multiple UEs. The lower tier consists of UEs, where the resources are most limited, and the applications require processed data. The second tier consists of edge servers deployed along with cellular towers, containing more resources as compared to the UEs, and routers, which are required to transfer and receive data from the cloud and have no processing resources of their own. The upper tier consists of cloud servers, which have a greater amount of resources as compared to the rest, because of which their resources are considered to be unlimited. The distance between the second and last tier is larger as compared to the distance between UEs and the second tier. The difference in distances is utilized to prioritize the task offloading in PHyPO.

To resolve the aforementioned problems, a comprehensive algorithm is formulated that uses the existing hardware components and provides solutions for the mentioned problems by use of the latest ML trends. A data-oriented optimal partitioning scheme is introduced, along with the utilization of an ensemble model, namely Adaptive Boosting (AdaBoost) for the reduction of computational complexity and prediction time. The use of Automated Machine Learning (AutoML) resolves the issue of selecting an optimal ML model for the given data, along with tuning the hyper-parameters until it finds global minima. The contributions of the proposed algorithm can be summed up as follows.

1. Development of an optimal task partitioning algorithm to minimize the cost before offloading.
2. Use of a 3-tier architecture to increase the service rate, by offloading time-sensitive tasks only on the nearby edge servers and the rest on either of the devices.
3. Use of AutoML to predict the optimal ML algorithm along with tuning its hyper-parameters.
4. Use of AdaBoost to eradicate the need for exhaustive search and optimize optimal partitioning and offloading problems in *O*(1) complexity.

### Paper organization

The rest of the paper is organized as follows. Second section contains the literature review, which is followed by the proposed methodology in the third section. Fourth section illustrates the results and the fifth section contains the conclusions along with the future research directions.

## Literature review

Wu et al. [21] generated an optimal task partitioning model for MCC. Using a graph-based algorithm, energy consumption along with delay was minimized for different environmental conditions, such as changes in bandwidth and server performance. The major drawback of this model is its complexity, which is more than *O*(*n*^2^). A Genetic Algorithm (GA) based algorithm was developed by Goudarzi et al. [22] to divide a task into an optimal number of partitions for offloading purposes. Data collected from real mobile applications were used to monitor the trends of data dependency. Each partitioning scheme of an application was represented by a single chromosome, which was to be executed in MCC. The model was evaluated based on the energy consumption, the total delay of the system, and the overall cost of execution. Partial offloading was implemented using the crossover characteristic of the GA. It is a stationary model with relatively less complexity, but more than *O*(1). The following subsections provide insight into the existing literature regarding computational offloading in MC.

### Algorithms for optimal offloading without learning

Kao et al. [23] developed a model for optimal offloading policy, consisting of a network with all the UEs connected via a direct link. In run-time, each UE was considered a node and when an application started, the node with resource demand was made a leader node. The purpose of the leader node was to collect information regarding the resources of all nearby nodes and then predict where the data was to be offloaded. The model was evaluated based on CPU performance and latency of task execution. However, this model has a high probability of resource contention. A context-aware offloading scheme was introduced in MCC by Chen et al. [24], where the cost function depended upon the services provided by the resources, and a significant amount of execution time and power consumption were reduced. The main limitations of this model are its 2-tier architecture and high complexity. Tran et al. [25] modeled an algorithm to offload data as a service in a fog architecture comprising many devices connected on the Internet of Things (IoT) treated as edge. Chang et al. [26] proposed a fog architecture-based model, but with energy-efficient optimization at UE. These models provide sub-optimal results, with more time complexity.

Maio et al. [27] proposed a mobile-edge-cloud-based model on simulated data to determine optimal offloading schemes, based on the preference of the user. Monte-Carlo simulations were also carried out to evaluate the proposed methodology, where to save the battery and lessen the application’s run-time, the model traded off tasks between the edge server and cloud. This model is also stationary, and the results are produced with relatively high computational complexity. A dynamic programming-based optimal scheduling and offloading model in MEC was developed by Hong et al. [28], which scheduled and offloaded tasks based on the priority of the user. Based on the tendency of a user, the model traded off between energy and latency. Additionally, the scheduling of tasks depended on the resources available in the MEC. The complexity of this model increases exponentially with the increase in task size, which makes it unsuitable to be used for larger task sizes. Another model was proposed by Chen et al. [29] in which the application of MEC in IoT was introduced for the development of a stochastic optimization algorithm for minimal energy consumption during transmission. It is an energy-oriented model with polynomial time complexity.

A model to secure the data on the physical layer during offloading in IoT-edge computing was developed by Liu et al. [30], using blockchain, which also worked on computational offloading. The resources with higher utility were given priority, and energy efficiency was also considered. However, this model is a binary offloading model with a complexity of more than *O*(1). Zhou et al. [31] proposed a reverse auction-based computation offloading and resource allocation mechanism for the mobile-edge-cloud computing architecture, with polynomial computational complexity.

### Algorithms for optimal offloading with machine learning

Researchers have worked on various unsupervised ML algorithms for optimal offloading scheme development. An energy-aware offloading scheme was developed by Xu et al. [32], which determined the nearest offloading node to the UE to consume less energy in transferring the data. Offloading time was optimized using GA, along with optimizing the energy consumption of all nodes. However, this model has a low convergence rate to the solution. The reliability of data in MEC for software-defined network-based internet of the vehicle was improved by Hou et al. [33] by Particle Swarm Optimization (PSO) based computational offloading. Another PSO-based model for the optimization of sub-carrier allocation was developed by Dai et al. [34]. The research focused on the number of resource-demanding devices in the network (i.e., UEs and IoT devices), and increasing the rate of service provided by the edge server. The model also resulted in generating the optimal policy in a lesser number of iterations. Kuang et al. [35] developed a task offloading algorithm in edge computing for smart IoT, and to reduce complexity, a GA-based model was developed.

Wang et al. [36] proposed a mechanism to reduce the workload during the offloading of tasks and optimize the performance of edge servers by formulation of a utility function, based on GA. A multi-problem scenario was solved by a model called BeCome by Xu et al. [37] to ensure data integrity by blockchain, optimal offloading, and resource allocation strategy generation via GA. Yang et al. [38] introduced an offloading time optimization scheme using a stochastic technique of the Markov Decision Process (MDP). This scheme was applied in the MEC. A Bayesian Learning Automata (BLA) based scheme for computational offloading in MEC was developed by Farahbaksh et al. [39]. Along with energy consumption and latency improvements, this model improved network usage by managing traffic. However, these models produce binary offloading decisions, along with high complexity.

Using supervised ML, Huang et al. [40] proposed a distributed Deep Learning (DL)-based model to optimize the binary offloading scheme in MEC. A gradient descent algorithm was also applied to optimize the input parameters of different DL models to improve the efficiency of the system. This model is application-constrained and only works better for gaming, AR, and VR applications, along with media services. Wu et al. [41] proposed an optimal MEC offloading model in a vehicle-to-vehicle network. To incorporate the issues raised by the mobility of vehicles, a Support Vector Machine (SVM) based model was developed that increased the speed of offloading by using roadside units for transfer purposes. With a time constraint, each server had to finish executing the task and send the data back to the vehicle. This model has more offloading overhead.

Sun et al. [42] developed a DL-based transfer learning model for optimal code computing in industrial IoT to improve service accuracy while minimizing latency. This model was centralized as it was deployed on an edge server. Hence, it increases the chances of the whole network malfunctioning in the event of any error on the edge server. Moreover, this model has a large overhead, which was solved by Ali et al. [43]. The authors in [43] developed an energy-efficient offloading scheme for optimal offloading in MEC, with a fixed number of partitions. To reduce the computational overhead, the data was generated for different iterations, and a model was trained using a supervised classifier of a Deep Neural Network (DNN), which resulted in an average accuracy of 70%.

Abbas et al. [20] proposed energy and latency optimizing cost function using UE and edge server architecture, for partial computational offloading. A DL technique was also applied with the resulting accuracy of 71%. Irshad et al. [44] proposed a time allocation scheme along with reducing the energy consumption of UE using a hybrid access point to connect to the MEC. This scheme also used a DL model and achieved an accuracy of about 72%. Shahidinejad et al. [45] developed a cloud and edge server-based model for mobile computing in which a cost function was generated using latency, energy, and the power utilization of the UE. The results showed an improvement in the network availability and the data was also secured using a federated learning framework.

An improved version of [43] was developed by Ali et al. [46] where different delays were considered for task offloading in MEC along with a DNN algorithm with a faster prediction speed in comparison with the previously developed models. These prediction models use the architecture of MEC and provide a binary offloading scheme, i.e., the subtask is offloaded only on the edge server or processed on the UE. There is more classification error in each model, except [45], but this model has more complexity as compared to the others, along with providing no solution for the time-sensitive applications. An edge-cloud-based offloading strategy was also developed by Mao et al. [47] where a DNN-based algorithm was used to optimize the energy consumption and reduce the latency for 6G-IoT devices.

Xu et al. [48] proposed a Reinforcement Learning (RL) based algorithm for the problems of offloading and auto-scaling in an energy-harvesting MEC. The data was trained for multiple iterations and compared with the Q-learning algorithm where the RL model learned better than the former model, in terms of the optimal cost function, and power consumption of the base station, along with average time consumption. This study does not consider the load balancing problem, which was resolved by Dinh et al. [49], who developed an RL-based offloading method in MEC to learn the pattern of offloading for known Channel State Information (CSI) and then applied it to all unknown CSIs of different games. The main constraint of this model is its binary offloading policy.

Zhang et al. [50] proposed a model for computational offloading in MEC based on Deep Q-Learning (DQL) along with an offloading algorithm to improve reliability in case of data transmission failure. Liu et al. [51] introduced a vehicular mobile-edge server-based architecture and by generating the data through stochastic routing, the computational offloading problem was learned and modeled by a DQL method, and a Deep Reinforcement Learning (DRL) model was proposed for the resource allocation problem. Both of the models are highly computationally complex. Min et al. [52] proposed an energy harvesting-based offloading scheme in MEC for IoT devices, using RL for online learning. Given the trained data, the model predicted an optimal partial offloading policy in run-time along with the offloading rate. In addition to the limitation of binary offloading, this model has high complexity.

Huang et al. [53] formulated a model for optimal task offloading in a dense network, along with optimizing the bandwidth allocation to each of the UEs in MEC. The model used an offline DQL algorithm to learn a nearly optimal model after around 8000 iterations. This model is centralized and does not apply to all types of applications. Yu et al. [54] proposed a Deep Imitation Learning (DIL) based model for optimizing the offloading policy with a fixed number of partitions in a mobile-edge-cloud server-based architecture. A large amount of data was generated to incorporate the changes in environmental conditions, and then DIL learned and cloned those changes to provide the classification label. This model was application-oriented, and to make its application more vast, a multi-problem-solving model was developed by Yu et al. [55], which used the DRL algorithm for offloading and resource allocation in MEC. Liao et al. [56] proposed an algorithm to offload tasks with priority to the edge servers. The proposed double reinforcement learning computation offloading algorithm not only makes the offloading decision but also predicts the transmit power and the CPU frequency required. This complex model is deployed on UE, and handles the tasks with high priority only, while the rest are processed on the UE. Mao et al. [57] also proposed an algorithm to jointly optimize energy and latency during task offloading for V2V network using DQL, which resulted in an improved performance in terms of average task completion ratio. Table 1 summarizes the related work presented in this section.

**Table 1:** Comparison of related work.

Ref #	Year	Energy	Delay	Partition Scheme	Dataset	ML Model	Network
Wu et al. [21]	2016	✓	✓	✓	Simulated	N/A	Sparse
Goudarzi et al. [22]	2017	✓	✓	✓	Real	GA	Sparse
Kao et al. [23]	2017	×	✓	✓	Real	N/A	Dense
Xu et al. [48]	2017	✓	✓	×	Simulated	RL	Dense
Chen et al. [24]	2017	✓	✓	✓	Real	N/A	Sparse
Tran et al. [25]	2017	×	✓	×	Real	N/A	Dense
Chang et al. [26]	2017	✓	×	×	Simulated	N/A	Dense
Dai et al. [34]	2018	✓	✓	×	Simulated	PSO	Dense
Dinh et al. [49]	2018	✓	×	×	Real	RL	Dense
Maio et al. [27]	2018	✓	✓	×	Simulated	N/A	Sparse
Huang et al. [40]	2018	✓	✓	×	Simulated	DL	Sparse
Wu et al. [41]	2018	×	✓	×	Simulated	SVM	Sparse
Hong et al. [28]	2019	✓	✓	×	Simulated	N/A	Sparse
Zhang et al. [50]	2019	×	✓	×	Real	DQL	Dense
Liu et al. [51]	2019	×	✓	×	Simulated	DQL & DRL	Dense
Chen et al. [29]	2019	✓	×	×	Simulated	N/A	Sparse
Sun et al. [42]	2019	×	✓	×	Real	DL & TL	Dense
Ali et al. [43]	2019	✓	✓	×	Simulated	DL	Sparse
Min et al. [5]	2019	✓	✓	×	Simulated	RL	Sparse
Xu et al. [32]	2019	✓	✓	×	Simulated	GA	Dense
Huang et al. [53]	2019	✓	✓	×	Simulated	DQL	Dense
Yu et al. [54]	2020	✓	✓	×	Simulated	DIL	Sparse
Hou et al. [33]	2020	×	✓	×	Simulated	PSO	Dense
Kuang et al. [35]	2020	✓	✓	×	Simulated	GA	Dense
Wang et al. [36]	2020	✓	×	×	Simulated	GA	Dense
Xu et al. [37]	2020	✓	✓	×	Simulated	GA	Dense
Abbas et al. [20]	2021	✓	✓	×	Simulated	DL	Sparse
Irshad et al. [44]	2021	✓	✓	×	Simulated	DL	Sparse
Yang et al. [38]	2021	×	✓	×	Simulated	MDP	Dense
Yu et al. [61]	2021	✓	×	×	Simulated	DRL	Dense
Shahidinejad et al. [45]	2021	✓	✓	×	Simulated	FL	Dense
Ali et al. [46]	2021	✓	✓	×	Simulated	DL	Sparse
Farahbaksh et al. [39]	2021	✓	✓	×	Simulated	BLA	Dense
Mao et al. [47]	2021	✓	✓	×	Simulated	DNN	Dense
Liu et al. [30]	2022	✓	×	×	Simulated	N/A	Dense
Zhou et al. [31]	2022	×	✓	×	Simulated	N/A	Dense
Liao et al. [56]	2022	✓	✓	×	Simulated	RL	Sparse
Zhang et al. [58]	2023	✓	✓	×	Real	N/A	Dense
Mao et al. [57]	2024	✓	✓	✓	Simulated	DQL	Dense
PHyPO (Proposed)	2024	✓	✓	✓	Simulated	Ensemble	Dense

## PHyPO

The communication and processing parameters of a UE may vary due to the changing amount of available resources like CPU frequency, sub-carriers of the channel, and the location of the UE. A comprehensive cost function is formulated to compare different offloading schemes based on two parameters, i.e., latency and energy consumption. A 3-class dataset is generated and classified using different ML models. The comparison is made with the most commonly used ML models.

### Proposed architecture

The proposed architecture consists of a 3-tier architecture, which includes UEs, edge servers, and cloud servers, as shown in Fig 1. The communication between UEs and edge servers is carried out directly, while cloud communication is carried out via a router. The time sensitivity of a task is also considered using a parameter of priority. The tasks with high time sensitivity are given priority over other tasks. As the latency for cloud communication is high due to the distance between UE and the cloud, for high-priority tasks, PHyPO considers the offloading scheme using only UE and edge resources. For the low-time sensitivity tasks, the offloading architecture includes all the components of the system model. This hybrid scheme increases the service rate too, resulting in utilizing the least amount of resources of the UE. For task partitioning, an already established partitioning scheme of ^*a*^*Z_b_* is used, where *a* is the task size and *b* is the number of components it is divided into [43]. In this scheme, the computational complexity of task partitioning is reduced by limiting the number of components along with defining the resolution of partitioning. Table 2 contains a summary of notations used in this paper, along with their descriptions.

**Table 2:** List of notations.

Notations	Description
*b*_*e*_	Bandwidth for communication between UE and edge server
*c*	Speed of light
*d*	Distance between UE and edge server
*f*_*em*_	CPU frequency of edge server to process *ST*_*m*_
*k*_*m*_	Number of sub-carriers available to send *ST*_*m*_ to edge and receive the processed task
*n*	Total number of sub-tasks
*m*	Index of sub-task
*p*	Priority of a task
*r*_*ac*_	Data transmission rate between cloud and router
*r*_*m*_	Number of CPU cores available to process *ST*_*m*_ at edge server
*s*_*m*_	Size of *ST*_*m*_ in MBs
*C*	Cost of execution
*E*	Energy
*E*_*cm*_	Energy consumed to process *ST*_*m*_ on cloud server
*H*	Number of CPU cycles per 1 MB
*K*	Total number of sub-carriers of a communication channel between UE and edge server
*L*	Latency
*P*	Power
*R*	Total number of CPU cores available at an edge server
*S*	Size of the complete task
*ST*_*m*_	Sub-task of *m*th index
*β*	Per unit cost of router usage
*ϵ*	Constant dependent on average switch capacitance and average activity factor of *UE*_*m*_
*δ*	Weighting coefficients for cost function
*θ*	Path loss exponent

### Latency of PHyPO

The latency of each sub-task for all schemes is calculated for the input parameters. The total latency includes the time taken to divide a task into sub-tasks, transmit it, and receive it from either of the servers and the time taken to process it.

For local execution of *ST*_*m*_, the time required to divide a task into *n* sub-tasks is given by

$$\begin{array}{c} L_{dm}=\frac{(n-1)\times \tau }{n}, \end{array}$$ (1)

where *τ* is the time required to divide a task into 2 sub-tasks [43]. After the division of the task, the local processing parameters are used to execute the sub-task, the time consumed during which is given by

$$\begin{array}{c} L_{im}=\frac{\alpha_{m}}{f_{cm}}, \end{array}$$ (2)

where *α*_*m*_ is the number of CPU cycles used by UE to process the sub-task and *f*_*cm*_ is the local CPU frequency [20]. The value of *α*_*m*_ can be calculated by

$$\begin{array}{c} \alpha_{m}=s_{m}\times \eta , \end{array}$$ (3)

where *η* is the number of CPU cycles required to process each MegaByte (MB) of the *ST*_*m*_ and *s*_*m*_ is the size of *ST*_*m*_ [44]. The total execution latency for local processing is calculated by

$$\begin{array}{c} L_{lm}=L_{dm}+L_{im}. \end{array}$$ (4)

The total latency of edge offloading is calculated by summing all the delays included in the total processing of *ST*_*m*_ as

$$\begin{array}{c} L_{em}=L_{tm}+L_{om}+L_{rm}+2L_{pm}+L_{dm}, \end{array}$$ (5)

where *L*_*om*_ and *L*_*rm*_ are times used by the UE to prepare the data for transmission to the edge server and to load the output data when the edge server sends it back, respectively [43]. *L*_*pm*_ the propagation delay for sending data to and from the edge server. It is added twice to incorporate both the delay for uplink and downlink communications. The value of total propagation delay is dependent on the medium of communication, which in this case is air, and is calculated as

$$\begin{array}{c} L_{pm}=\frac{d}{c}, \end{array}$$ (6)

where *d* is the distance between UE and edge, and *c* is the speed of light, as the medium of transportation is air. The time consumed by the transmission and reception is calculated, respectively, as

$$\begin{array}{c} L_{tm}=\frac{s_{m}}{\upsilon_{ulm}}, \end{array}$$ (7)

$$\begin{array}{c} L_{rm}=\frac{s_{m}}{\upsilon_{dlm}}, \end{array}$$ (8)

where *υ*_*ulm*_ and *υ*_*dlm*_ are the uplink and downlink data rates for communication between the UE and edge. The value of the uplink data rate is calculated as

$$\begin{array}{c} \upsilon_{upm}=\frac{k_{m}}{K}\times b_{e}\times {\text{log}}_{2}\left(\phi_{upm}\right), \end{array}$$ (9)

and the value of the downlink data rate is given by

$$\begin{array}{c} \upsilon_{dlm}=\frac{k_{m}}{K}\times b_{e}\times {\text{log}}_{2}\left(\phi_{dlm}\right). \end{array}$$ (10)

In (9) and (10), *b*_*e*_ is the bandwidth of the channel between UE and the edge server. The transmission and reception of data are directly proportional to the number of sub-carriers available for communication. *ϕ*_*upm*_ and *ϕ*_*dlm*_ are coefficients that are used for simplification purposes and their values are stated, respectively, as

$$\begin{array}{c} \varphi_{upm}=1+\frac{P_{tm}\times {\left|h_{up}\right|}^{2}}{\Gamma \left(g_{up}\right)\times d_{m}^{\theta }\times N_{o}}, \end{array}$$ (11)

$$\begin{array}{c} \varphi_{dlm}=1+\frac{P_{rm}\times {\left|h_{dl}\right|}^{2}}{\Gamma \left(g_{dl}\right)\times d_{m}^{\theta }\times N_{o}}, \end{array}$$ (12)

where *N*_*o*_ is the noise power, while *P*_*tm*_ and *P*_*rm*_ are the values of power used to transmit *ST*_*m*_ to the edge server and to receive it back, respectively. The communication channel used is the Rayleigh channel, so the channel fading coefficient for uplink is calculated by

$$\begin{array}{c} h_{up}=\sqrt{d^{-\theta }}\times \left[N\left(0,1\right)+jN\left(0,1\right)\right], \end{array}$$ (13)

where *N* is generated as a random Gaussian variable between 0 and 1. In this model, the channel fading coefficient for the downlink is considered to be equal to the uplink fading coefficient. The Signal-to-Noise Ratio (SNR) margins that are used to synchronize the actual signal using the bit error rate, i.e., *g*_*up*_ and *g*_*dl*_ for uplink and downlink channels, respectively, are calculated as

$$\begin{array}{c} \Gamma \left(g_{up}\right)=\frac{-2\text{log}(5g_{up})}{3}, \end{array}$$ (14)

$$\begin{array}{c} \Gamma \left(g_{dl}\right)=\frac{-2\text{log}(5g_{dl})}{3}. \end{array}$$ (15)

The time required to process data at the edge server is calculated using the processing frequency of the edge server, *f*_*em*_, and the number of CPU cores available, as

$$\begin{array}{c} L_{om}=\frac{\alpha_{m}}{r_{m}\times f_{em}}. \end{array}$$ (16)

The total latency in the case of cloud computing is the cumulative time taken to send the data to the router, which sends it to the cloud server, and then the same data transfer occurs in the downlink. The overall latency of mobile-cloud computing is calculated as

$$\begin{array}{c} L_{acm}=L_{am}+L_{cm}+L_{dm}, \end{array}$$ (17)

where *L*_*am*_ is the latency to send the sub-task to the router in uplink and then receive the processed task back from it. *L*_*am*_ is calculated as

$$\begin{array}{c} L_{am}=\frac{u_{inm}}{\eta_{m}^{u}\times b_{m}^{u}}+\frac{u_{outm}}{\eta_{m}^{d}\times b_{m}^{d}}, \end{array}$$ (18)

where *u*_*inm*_ and *u*_*outm*_ are the input and output sizes of the sub-task, which are considered to be equal to the size of *s*_*m*_ in MBs [59]. $\eta_{m}^{u}$ and $\eta_{m}^{d}$ represent the uplink and downlink transmissions’ spectral efficiency, respectively. The delay in sending the task from the router to the cloud and then receiving the processed task at the UE is calculated as

$$\begin{array}{c} L_{cm}=\frac{L_{am}}{r_{ac}}, \end{array}$$ (19)

where *r*_*ac*_ is the rate of data transmission between the cloud and the UE [59].

### Energy consumption of PHyPO

The energy consumption of each scheme is calculated using the different parameters involved in each scenario. For local execution, the total energy is calculated as

$$\begin{array}{c} E_{lm}=E_{im}+E_{dm}. \end{array}$$ (20)

In (20), the total energy utilized in the local execution of *ST*_*m*_ is calculated by adding the energy utilized in the processing of *ST*_*m*_, i.e., *E*_*im*_ and partitioning of the whole task [43], i.e. *E*_*dm*_, which is calculated as

$$\begin{array}{c} E_{dm}=L_{dm}\times \epsilon \times f_{cm}^{3}, \end{array}$$ (21)

and the value of *E*_*im*_ is calculated as

$$\begin{array}{c} E_{im}=L_{im}\times \epsilon \times f_{cm}^{\zeta }, \end{array}$$ (22)

where *ζ* is a constant of the UE, having a value greater than 2 [43]. In the case of edge computing, the energy required to transmit data to the edge server from the UE is calculated as

$$\begin{array}{c} E_{tm}=L_{tm}\times P_{tm}, \end{array}$$ (23)

and the energy used to receive the data from the edge server is calculated as

$$\begin{array}{c} E_{rm}=L_{rm}\times P_{rm}, \end{array}$$ (24)

where *P*_*tm*_ and *P*_*rm*_ are the values of power consumption of the UE for transmitting the data to the edge server and then receiving from it, respectively. The total energy consumed in edge offloading is measured by the sum of *E*_*tm*_ and *E*_*rm*_ [43], and is given as

$$\begin{array}{c} E_{em}=E_{tm}+E_{rm}+E_{dm}. \end{array}$$ (25)

As the energy used by the router to transmit the data to the cloud has no impact on the battery of the UE, so in the case of cloud computing, only the energy consumption of the UE to transmit *ST*_*m*_ to the router is considered [59]. Therefore,

$$\begin{array}{c} E_{cm}=E_{ctm}+E_{crm}+E_{dm}+\left(\beta \times s_{m}\right), \end{array}$$ (26)

where *β* is the per MB cost of router usage, *E*_*ctm*_ and *E*_*crm*_ are the energy values of the UE used to transmit the data to the router and then receive the processed data.

### Cost functions of PHyPO

A cost function is generated using the parameters of latency and energy consumption, which are normalized to make them unitless quantities and then weighted with respect to their effect on the whole function. The following expressions are the cost functions for local execution, edge, and cloud server offloading, respectively.

$$\begin{array}{c} C_{lm}=\left(\delta_{1}\times \frac{L_{lm}}{L_{max}}\right)+\left(\delta_{2}\times \frac{E_{lm}}{E_{max}}\right), \end{array}$$ (27)

$$\begin{array}{c} C_{em}=\left(\delta_{3}\times \frac{L_{em}}{L_{max}}\right)+\left(\delta_{4}\times \frac{E_{em}}{E_{max}}\right), \end{array}$$ (28)

$$\begin{array}{c} C_{cm}=\left(\delta_{5}\times \frac{L_{acm}}{L_{max}}\right)+\left(\delta_{6}\times \frac{E_{cm}}{E_{max}}\right), \end{array}$$ (29)

where *δ*_1_, *δ*_2_, …, *δ*_6_ are the weighting coefficients of cost functions for different schemes, i.e. *C*_*lm*_, *C*_*em*_, and *C*_*cm*_. Individual costs of each sub-task are calculated and then for each possible policy of offloading, the total cost of executing the task is calculated, e.g., if a task has 2 partitions, the total possible number of offloading policies can be 9 as there are 3 destinations in this case. For each possible offloading policy, the cumulative cost is compared with the rest, and the minimum one is chosen for the processing. The offloading policy and the number of partitions in case of minimum cost are regarded as the optimal policies against the particular input variables.

### Machine learning in PHyPO

**Fig 2:**
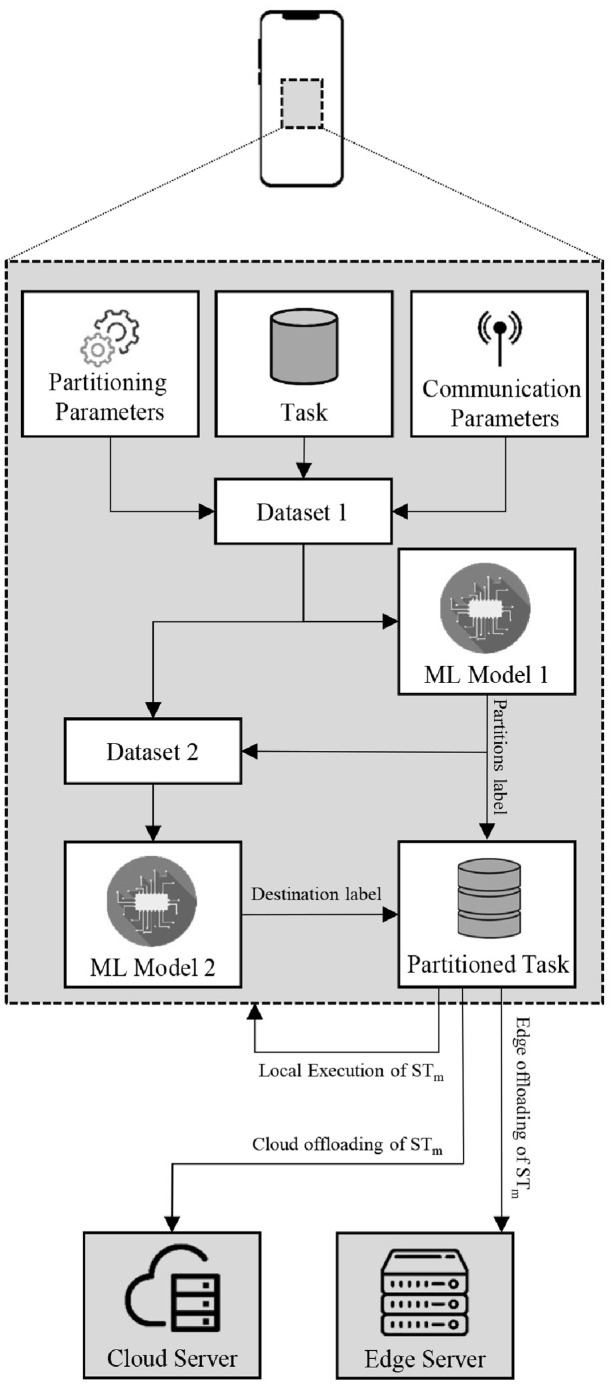
An overview of PHyPO.

The calculation of cost for each possible offloading policy costs a lot of time and battery consumption, so to eradicate the need for an exhaustive search, ML models are generated. The data is trained for supervised learning with AutoML, having a classification label being the optimal number of partitions and offloading policy, as mentioned in the previous section. AutoML is the latest trend in the field of ML, which is used to determine which ML model is optimal for the classification of any given data [60]. There are several parameters that an ML model sets during the training process, but some are set before the beginning of the training process. These parameters are called hyper-parameters, which also play a drastic role in changing the classification value. AutoML not only predicts the optimal ML model but also sets the value of hyper-parameters for the model by tuning them in different iterations until no further improvement in the results can be achieved. This process is not only efficient but also eliminates the chances of human error in the identification and tuning of an ML model [60]. By implementation of the ML models, the complexity of generating output is reduced to *O*(1), as the model is trained only once. As an ML classifier only provides a single class label, 2 models are generated to optimize the number of partitions and their offloading destination. Fig 2 shows a brief overview of the PHyPO algorithm, which is deployed on the UE.

The datasets are trained using the AutoML generated model and then compared with multiple traditional ML models, which include Tree, K-Nearest Neighbor (KNN), SVM, Neural Network (NN), and ensemble models. These models are trained once and then deployed for run-time testing with minimum time and energy consumption [61, 62]. The first dataset (DS1) is generated by concatenation of the total task size, the communication parameters used to communicate with the edge server, and the parameters used in local processing. The local processing parameters include the CPU frequency of UE and the resolution with which the task is to be partitioned, and both of them vary with respect to the time and position of the UE. The communication parameters include the distance between the UE and the edge server, the number of sub-carriers, and the number of CPU cores available at the edge server. The second dataset (DS2) is generated by concatenation of DS1 and the label generated by the DS1, in case of testing. The task is partitioned with the label generated by Model 1 and then the partitioned sub-tasks are offloaded to their respective targets using the label generated by Model 2. Both the datasets used to generate the test label for sub-task *m* can be generalized by the following equations

$$\begin{array}{c} DS1_{iteration}=\left[S,p,f_{cm},d,r_{i},k_{i},resolution\right], \end{array}$$ (30)

$$\begin{array}{c} DS1_{iteration}=\left[S,p,f_{cm},d,r_{i},k_{i},resolution,label1\right]. \end{array}$$ (31)

### Use of ensemble learning

**Fig 3:**
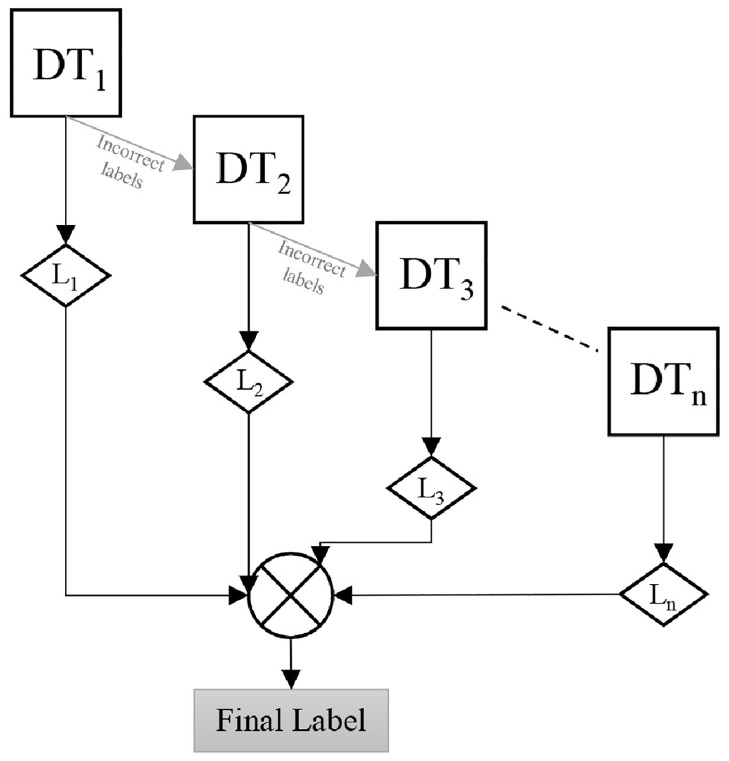
The basic working of AMP and AMO.

The ensemble learning models work by stacking the output of multiple weak classifiers and then choosing the most frequent output. Fig 3 shows the basic working of the AdaBoost model, which in this case is classified using a series of Decision Trees (DTs). An ensemble model can either be implemented by using Bagging-based models or Boosting-based models. The PHyPO algorithm utilizes a Boosting-based model for two purposes: (i) AdaBoost Model for Partitioning (AMP) and (ii) AdaBoost Model for Offloading (AMO). An AdaBoost model works by training the data on a weak classifier and then passing on its misclassified data to the next classifier, which then passes on its misclassified labels to the next classifier and so on [63].

Algorithm 1 depicts the pseudocode of the PHyPO algorithm with the adaptive boosting training models of AMP and AMO, along with the process of generation of the datasets. Both of these algorithms are tuned with different hyper-parameters and are compared with other ensemble models as well, which include Subspace KNN, Bagging Trees, and other variants of Boosting Trees.

After the deployment of the trained models on the UE, Algorithm 1 is used to generate the label and perform the action upon it.

**Algorithm 1** PHyPO scheme in mobile computing

Input: *S*, *resolution*, *MaxComponents*

Output: *label*1, ***label2***, *y*_1_, *y*_2_

1. ***PD*** =^*a*^ *Z*_b_*Partitioning*(*S*, *resolution*, *MaxComponents*)

2. **For** *iteration* = 1 *to* 1000

3.  *P* = *GenerateRandomInteger* [0, 1]

4.  *f*_*cm*_ = *GenerateRandomInteger* [10^8^, 10^9^]

5.  *d* = *GenerateRandomInteger* [20, 35000]

6.  ***LC*** = *CalculateLocalCost*(***PD***, *f_cm_*)

7.  [***EC***, ***r***_*i*_, ***k***_*i*_] = *CalculateEdgeCost*(***PD***, *d*, *f_cm_*)

8.  ***CC*** = *CalculateCloudCost*(***PD***, *f_cm_*)

9.  [*row*, *col*] = *Size*(***PD***)

10.  **For** *k* = 1 *to row*

11.   **if** *P* = 0

12.    ***ALL_COST*** = [***LC***(*k*), ***EC***(*k*), ***CC***(*k*)]

13.   **else**

14.    ***ALL_COST*** = [***LC***(*k*), ***EC***(*k*)]

15.   **End if**

16.   ***CMB_COST*** = *AllCombinations*(***ALL_COST***)

17.   ***temp*** = *Sum*(***CMB_COST***(*Rows*))

18.   *index* = *Min*(***temp***)

19.   ***TEMP2*** = ***V***(*index*)

20.   **For** *j* = 1 *to col*

21.    **if** ***TEMP2***(*k*, *j*) = ***LC***(*k*, *j*)

22.     *OP* = 1

23.    **else if** ***TEMP2***(*k*, *j*) = ***EC***(*k*, *j*)

24.     *OP* = 2

25.    **else if** ***TEMP2***(*k*, *j*) = ***CC***(*k*, *j*)

26.     *OP* = 3

27.    **End if**

28.   **End For**

29.  **End For**

30.  ***temp3*** = *Sum*(***TEMP2***(*Rows*))

31.  [*MinCost*, *index2*] = *Min*(***temp3***)

32.  ***label2***(*iteration*) = ***OP***(*index2*)

33.  *label*1 = *Length*(*label*2)

34.  ***DS1***(*iteration*) = [*S*,*P*,*f*_*cm*_,*d*,*r*_*i*_,*k*_*i*_,*resolution*,*label*1]

35.  ***DS2***(*iteration*) = [*S*,*P*,*f*_/_*cm*,*d*,*r*_*i*_,*k*_*i*_,*resolution*,*label*1, ***label2***]

36. **End For**

37. *AMP* = *AutoML*(***DS1****_train_*)

38. *AM0* = *AutoML*(***DS2****_train_*)

39. *y*_1_ = *Test*(*AMP*(***DS1****_test_*))

40. *y*_2_ = *Test*(*AMP*(***DS2****_test_*)) Here, *S* is the size of the task, *resolution* is the size of a minimum possible partition, and *MaxComponents* is the number of components the task is decomposed into. *y*_1_ is the test label of the ML model *AMP* and *y*_2_ is the test label of *AMO*, both of which are determined by training both the datasets, **DS1**_*train*_ and **DS2**_*train*_, in AutoML.

## Performance evaluation

The models are evaluated on 2 datasets, both generated by simulation. The simulation was carried out on MATLAB R2021a, with the system specifications being an Intel processor of Core-i7 and 8 GB of RAM, and a 64-bit operating system. The dataset was trained on multiple traditional ML models using the Classification Learner Application, the results of which were compared with the model generated by AutoML, giving the most accurate results.

### Dataset description

A multi-user scenario was considered where each UE could move within the range of ±100 meters during the processing of sub-tasks. The CPU frequency of UE ranged from 0.1 to 1 GHz and different task sizes were considered, ranging from 100 MB to 2 GB with different partitioning resolutions. For communication between UE and the edge server, a 4G band was used and the value of the distance between UE and the edge server varied from 20 meters to 35 km. As the edge server resources are limited, the possibility of 0 CPU cores available at the edge server was also considered. To generate a comprehensive dataset that incorporated each possible input scenario, the optimal cost for each task size was generated multiple times.

### Evaluation parameters

The performance of the proposed algorithm was evaluated on the basis of the normalized cost of different execution schemes, testing accuracy of ML models, prediction time of the ML models, and the computational complexity of the model. The execution schemes include the proposed algorithm, total local execution, total edge server offloading, and total cloud server offloading. Table 3 shows the values of the parameters that were fixed before the simulations were carried out. The values of these parameters were determined by using 4G as a communication band for the communication between the edge server and UE. For communication between UE and the cloud server, the communication parameters used in WiFi were used.

**Table 3:** Configuration of simulation parameters.

Parameter	Configuration
*b*_*e*_	5 MHz
$b_{m}^{u}$ & $b_{m}^{d}$	2 GHz
*f*_*em*_	10 GB
*g*_*up*_ & *g*_*dl*_	1.25 × 10^−10^ MB
*k*_*m*_	1 − 256
*r*_*ac*_	0.1 MB/s
*r*_*m*_	0 − 16
*K*	256
*N*_*o*_	3.98 × 10^−21^ W/Hz
*P*_*tm*_ & *P*_*rm*_	0.8 to 1.25 W
*R*	16
*β*	0.08
*δ*_1_, *δ*_2_	0.6,0.4
*δ*_3_, *δ*_4_	0.6,0.4
*δ*_5_, *δ*_6_	0.7,0.3
*ϵ*	10^−27^
*ζ*	3
*η*	590 × 10^7^ cycles/MB
$\eta_{m}^{u}$ & $\eta_{m}^{d}$	3.98 × 10^−8^ MB/s/Hz
*θ*	3
*τ*	10^−3^ s

### Performance of cost function for time-insensitive tasks

**Fig 4:**
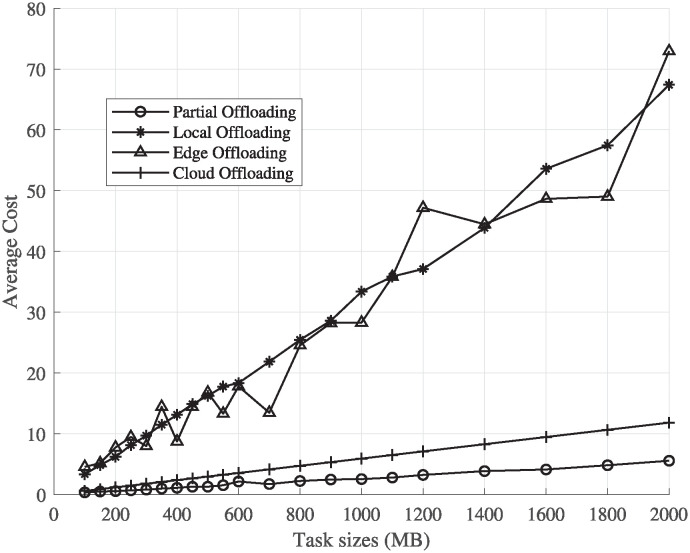
Performance evaluation of different schemes for time-insensitive tasks.

The average cost of PHyPO for each task size is compared with the cost of executing the whole task on UE or offloading the whole task on the edge server or cloud server. Fig 4 shows the comparison between all the execution schemes, where despite the linear increase in the cost with respect to the task size, the average normalized cost of PHyPO is lesser than the other costs. The average cost of PHyPO is lesser than the rest, and the performance of cloud computing is better than the other schemes, as the bandwidth of the cloud communication channel is considered to be 4 times the one used to communicate with the edge. The cost of edge computing is affected by its dependency on the number of available sub-carriers and the available CPU cores, with the cost resulting in infinity in case of no availability of cores, which is the reason behind the increase in the average cost. The service rate is also increased as the load on the edge server is lessened with the inclusion of a cloud server in the system model.

### Performance of cost function for time-sensitive tasks

**Fig 5:**
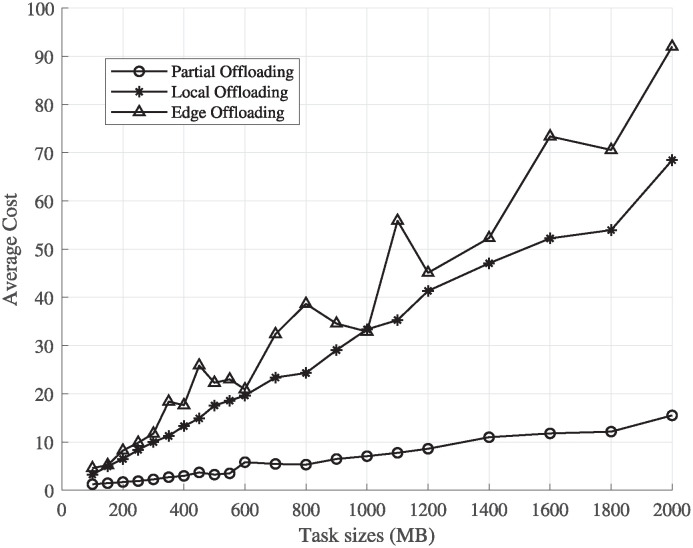
Performance evaluation of different schemes for time-insensitive tasks.

For time-sensitive tasks, the sub-tasks are offloaded only on the edge server or are executed locally, as cloud servers introduce more latency. Fig 5 shows the difference in average normalized cost of all three schemes, with a significant improvement in the PHyPO scheme results, as the latency and energy consumption both are reduced to a great extent. The great improvement in the performance of PHyPO in Fig 5 is also caused by the pre-calculation of an optimal number of partitions, as the number of components is not fixed in this model and each possible policy of partitioning a task is considered.

### AMP: AdaBoost based model for optimal partitioning

**Fig 6:**
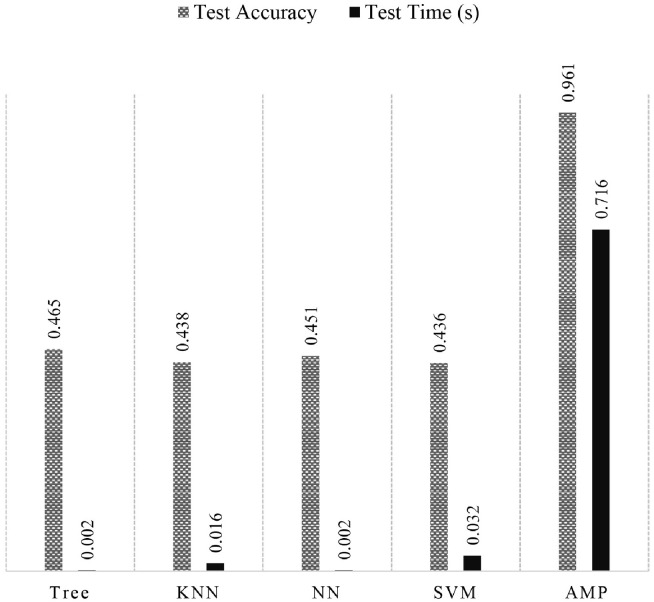
Performance of traditional ML algorithms for optimal partitioning scheme.

The first generated dataset is trained for multiple traditional ML models, which included Fine Tree, Coarse KNN, TriLayered NN, Fine Gaussian SVM, and an AutoML-tuned AdaBoost classifier. Fig 6 shows the comparison between all the mentioned models for the parameters of testing time and accuracy. The optimization time for an AMP is 0.7 seconds, which is relatively larger than the rest of the algorithms, but the reduction in error rate compensates for that. The performance of different bagging, boosting, and subspace ensemble methods were compared, and the best-performing ones are reported in Table 4. The proposed model, AMP, is also optimized via AutoML and tuned for various hyper-parameters for Bagging, RUSBoost, and AdaBoost Tree. Table 5 depicts the parameters that were tuned in different iterations to reduce the classification error rate.

**Table 4:** Performance of different ensemble algorithms for optimal partitioning policy.

	Training accuracy %	Testing accuracy %	Training time (s)	Testing time (s)
**Subspace KNN**	88.1	92.6	25.887	0.1020
**Bagged Tree**	64.9	71.0	50.895	0.1022
**AdaBoost (AMP)**	**91.4**	**96.1**	**703.6**	**0.7156**

**Table 5:** Tuning of hyper-parameters in each iteration for AMP.

Classification Error	Algorithm	No. of Learners	Learning rate	Max no. of splits	No. of predictors to samples
0.7314	RB	65	0.25673	54	N/A
0.5205	Bag	268	N/A	1	4
0.5205	AB	378	0.007298	265	N/A
0.4432	AB	46	0.18736	45478	N/A
0.2572	AB	317	0.001004	12181	N/A
0.0898	AB	378	0.28858	6764	N/A
0.0898	Bag	11	N/A	711	7
0.0898	RB	31	0.001252	1	N/A
0.0857	Bag	495	N/A	44447	3
0.0856	AB	467	0.4765	4545	N/A

Note: RB: RUSBoost, AB: AdaBoost, Bag: Bagging.

The training accuracy of the last 2 iterations in Table 5 has very little difference but due to the 44447 number of splits in the Bagging model, the testing time is much greater than the AMP. Any further tuning showed no improvement in the performance. Also, the reason behind a relative increase in the testing time of AMP than the rest of the ML models, as shown in Fig 6, is the greater number of learners than the other models having simple architectures, with the learners being DTs in this case.

### AMO: AdaBoost based model for optimal offloading

**Fig 7:**
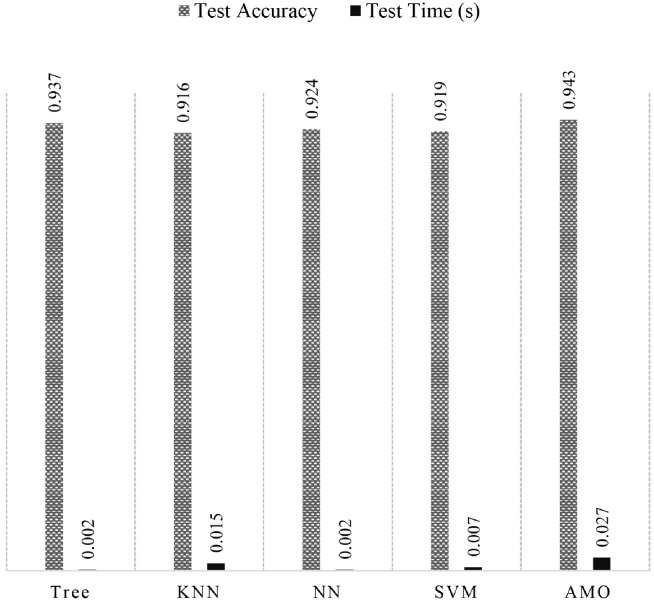
Performance of traditional ML algorithms for optimal offloading scheme.

The second dataset resulting from the concatenation of the first dataset and the output of AMP was also trained using AutoML, with the best results achieved by the AdaBoost model with differently tuned hyper-parameters, and compared with different conventional ML models. Fig 7 shows the comparison between Fine Tree, Coarse KNN, TriLayered NN, Fine Gaussian SVM, and the proposed AdaBoost-based Model for optimal Offloading (AMO). The performance of the proposed model AMO is seen to be better than the rest of the models with the least amount of classification error, along with the prediction time of 0.02 seconds, which is not much greater than the rest of the models. Due to a 0.6% error reduction in AMP as compared to Fine Tree, the latter is not considered the optimal model for offloading policy. Table 6 shows the comparison between different ensemble learning methods which included Subspace KNN, Bagging, and Boosting trees, while the best-performing ones are also mentioned in the table. The proposed model AMO to solve the second problem of optimal offloading policy performs better than the rest of the algorithms mentioned in Table 6 due to its simpler architecture and comprehensive dataset. This model is also optimized by tuning its hyper-parameters in various iterations along with the comparison with other ensemble models, which include Bagging, RUSBoost, and AdaBoost Tree, as shown in Table 7. The error reduction stopped after only a few iterations, from 6% to 5.8% error in prediction. The testing time of AMO is smaller than that of AMP due to the fewer number of learners and splits of the model.

**Table 6:** Performance of different ensemble algorithms for optimal offloading policy.

	Training accuracy %	Testing accuracy %	Training time (s)	Testing time (s)
**Subspace KNN**	88.1	92.6	25.887	0.1020
**Bagged Tree**	64.9	71.0	50.895	0.1022
**AdaBoost (AMP)**	91.4	96.1	703.6	0.7156

**Table 7:** Tuning of hyper-parameters in each iteration for AMO.

Classification Error	Algorithm	No. of Learners	Learning rate	Max no. of splits	No. of predictors to samples
0.06035	Bag	41	N/A	120	8
0.06035	RB	44	0.01012	19449	N/A
0.06035	AB	39	0.62092	587	N/A
0.06035	Bag	10	N/A	183	5
0.05834	Bag	11	N/A	708	9
0.05834	RB	46	0.001194	102	N/A
0.05834	AB	51	0.58222	41	N/A

Note: RB: RUSBoost, AB: AdaBoost, Bag: Bagging.

### Critical discussion

A novel algorithm to address the problem of data partitioning and offloading in MC is presented in this paper. A comprehensive dataset is simulated, containing different environmental scenarios, which is trained using an ML model to optimize the number of partitions and their offloading destinations, for time-sensitive tasks. From 100 MB to 1.2 GB, the dataset was generated for every task size with a gap of 50MB, but due to resource limitation, the gap between 1.2 GB to 2 GB was maintained as 100 MB, which is a limitation of our dataset. In our future work, we will seek to address this limitation by creating a model that trains itself in the run-time by using the test data of different sizes. Tasks are partitioned and offloaded without scheduling them first, which can be addressed in the future by scheduling them on the basis of priority and then partitioning them.

The parameters on which the proposed model is evaluated are average offloading energy consumption and latency, which are the most commonly used evaluation parameters and are also the indicator of quality of service in the literature. Thus, we have shown to achieve an overall higher quality of service. We aim to evaluate PHyPO on the basis of other evaluation parameters in the future, including task completion rate, and by using more resources for data training and testing. The technique of AutoML is used to determine the optimal model for the given data, along with the hyper-parameters. The training and testing accuracy of both the models, i.e., AMP and AMO, show that the data provided for training the model is comprehensive and contains all the relevant information required for prediction. The use of AdaBoost results in improved performance as it tunes the local parameters, which are used in each dataset, with respect to the weaknesses found in the previous one. The other hyper-parameters involved are tuned via AutoML to generate a model, which works optimally in each scenario. The prediction time of AMP is relatively high as compared to AMO due to more number of splits and number of learners. The data is tested in run-time using a distributed architecture, where with an insignificant increase in prediction time, near-optimal results are achieved, with the prediction accuracy of AMP being 96.1% and that of AMO being 94.3%. The achieved accuracy for both the algorithms is higher than the existing literature reviewed in the Literature review section, along with the proposed model being lightweight. We aim to improve this accuracy further in the future by performing data analysis and exploring other ML algorithms. This research can also be extended with the implementation of such a model, which can not only do optimal offloading but also solve the issue of resource allocation. Other aspects to explore in our future work are to apply task scheduling [64] before offloading, and the identification of tasks that cannot be partitioned in order to offload them accordingly.

## Conclusion and future work

The PHyPO algorithm provides a comprehensive task partitioning and offloading scheme in MC. The proposed 3-tier architecture improves the utility of the system and is also able to process bigger tasks. Both problems are solved with the computational complexity of *O*(1), by identifying the optimal algorithms using AutoML. The dataset creation with only the relevant features also decreases the prediction time, along with improvement in the accuracy of PHyPO. This work can be extended by solving the problem of resource allocation along with the current problem, use of network bands of 5G and beyond to minimize the chances of bottleneck in the case of MEC. Transfer learning can be applied to the generated model to include the tested data in the training model to make it more comprehensive and adaptive to every environmental condition. Task scheduling can also be applied before offloading, to further reduce the latency and enhance the overall efficiency of the system. As the proposed model only works for offloading when the tasks can be partitioned, this work can be extended to also identify the tasks that cannot be partitioned and offload them accordingly.

## Supporting information

S1 Data (MAT)

S2 Data (MAT)

S3 Data (MAT)

S4 Data (MAT)

S5 Data (MAT)

S6 Data (MAT)

S7 Data (MAT)

S8 Data (MAT)

S9 Data (MAT)

S10 Data (MAT)

S11 Data (MAT)

S12 Data (MAT)

S13 Data (MAT)

S14 Data (MAT)

S15 Data (MAT)

S16 Data (MAT)

S17 Data (MAT)

S18 Data (MAT)

S19 Data (MAT)

S20 Data (MAT)

S21 Data (MAT)

## References

[1] Zhang X, Chen H, Zhao Y, Ma Z, Xu Y, Huang H, et al. Improving cloud gaming experience through mobile edge computing. IEEE Wireless Communications. 2019;26(4):178–183. doi: 10.1109/MWC.2019.1800440

[2] Erol-Kantarci M, Sukhmani S. Caching and computing at the edge for mobile augmented reality and virtual reality (AR/VR) in 5G. Ad Hoc Networks. 2018; p. 169–177. doi: 10.1007/978-3-319-74439-1_15

[3] Abbas N, Zhang Y, Taherkordi A, Skeie T. Mobile edge computing: A survey. IEEE Internet of Things Journal. 2017;5(1):450–465. doi: 10.1109/JIOT.2017.2750180

[4] Orsini G, Bade D, Lamersdorf W. Computing at the mobile edge: Designing elastic android applications for computation offloading. In: 2015 8th IFIP Wireless and Mobile Networking Conference (WMNC). IEEE; 2015. p. 112–119.

[5] Sun H, Yu Y, Sha K, Lou B. mVideo: edge computing based mobile video processing systems. IEEE Access. 2019;8:11615–11623. doi: 10.1109/ACCESS.2019.2963159

[6] Chen X. Semantic analysis of multimodal sports video based on the support vector machine and mobile edge computing. Wireless Communications and Mobile Computing. 2022;2022.

[7] Khan WZ, Ahmed E, Hakak S, Yaqoob I, Ahmed A. Edge computing: A survey. Future Generation Computer Systems. 2019;97:219–235. doi: 10.1016/j.future.2019.02.050

[8] Cai W, Leung VC, Chen M. Next generation mobile cloud gaming. In: 2013 IEEE Seventh International Symposium on Service-Oriented System Engineering. IEEE; 2013. p. 551–560.

[9] Stantchev V, Barnawi A, Ghulam S, Schubert J, Tamm G. Smart items, fog and cloud computing as enablers of servitization in healthcare. Sensors & Transducers. 2014;185(2):121–128.

[10] Cao Y, Chen S, Hou P, Brown D. FAST: A fog computing assisted distributed analytics system to monitor fall for stroke mitigation. In: 2015 IEEE international conference on networking, architecture and storage (NAS). IEEE; 2015. p. 2–11.

[11] Taherkordi A, Eliassen F, Horn G. From IoT big data to IoT big services. In: Proceedings of the Symposium on Applied Computing; 2017. p. 485–491.

[12] Huang CM, Chiang MS, Dao DT, Su WL, Xu S, Zhou H. V2V data offloading for cellular network based on the software defined network (SDN) inside mobile edge computing (MEC) architecture. IEEE Access. 2018;6:17741–17755. doi: 10.1109/ACCESS.2018.2820679

[13] Ren J, He Y, Huang G, Yu G, Cai Y, Zhang Z. An edge-computing based architecture for mobile augmented reality. IEEE Network. 2019;33(4):162–169. doi: 10.1109/MNET.2018.1800132

[14] Liao B, Ali Y, Nazir S, He L, Khan HU. Security analysis of IoT devices by using mobile computing: a systematic literature review. IEEE Access. 2020;8:120331–120350. doi: 10.1109/ACCESS.2020.3006358

[15] Marotta MA, Faganello LR, Schimuneck MAK, Granville LZ, Rochol J, Both CB. Managing mobile cloud computing considering objective and subjective perspectives. Computer Networks. 2015;93:531–542. doi: 10.1016/j.comnet.2015.09.040

[16] Sahu I, Pandey U. Mobile cloud computing: Issues and challenges. In: 2018 International Conference on Advances in Computing, Communication Control and Networking (ICACCCN). IEEE; 2018. p. 247–250.

[17] Ahn S, Lee J, Park S, Newaz SS, Choi JK. Competitive partial computation offloading for maximizing energy efficiency in mobile cloud computing. IEEE Access. 2017;6:899–912. doi: 10.1109/ACCESS.2017.2776323

[18] Kai K, Cong W, Tao L. Fog computing for vehicular ad-hoc networks: paradigms, scenarios, and issues. the Journal of China Universities of Posts and Telecommunications. 2016;23(2):56–96. doi: 10.1016/S1005-8885(16)60021-3

[19] Kuang Z, Li L, Gao J, Zhao L, Liu A. Partial offloading scheduling and power allocation for mobile edge computing systems. IEEE Internet of Things Journal. 2019;6(4):6774–6785. doi: 10.1109/JIOT.2019.2911455

[20] Abbas ZH, Ali Z, Abbas G, Jiao L, Bilal M, Suh DY, et al. Computational offloading in mobile edge with comprehensive and energy efficient cost function: A deep learning approach. Sensors. 2021;21(10):3523. doi: 10.3390/s21103523 34069364 PMC8158712

[21] Wu H, Knottenbelt W, Wolter K, Sun Y. An optimal offloading partitioning algorithm in mobile cloud computing. In: International Conference on Quantitative Evaluation of Systems. Springer; 2016. p. 311–328.

[22] Goudarzi M, Zamani M, Toroghi Haghighat A. A genetic-based decision algorithm for multisite computation offloading in mobile cloud computing. International Journal of Communication Systems. 2017;30(10):e3241. doi: 10.1002/dac.3241

[23] Kao YH, Krishnamachari B, Ra MR, Bai F. Hermes: Latency optimal task assignment for resource-constrained mobile computing. IEEE Transactions on Mobile Computing. 2017;16(11):3056–3069. doi: 10.1109/TMC.2017.2679712

[24] Chen X, Chen S, Zeng X, Zheng X, Zhang Y, Rong C. Framework for context-aware computation offloading in mobile cloud computing. Journal of Cloud Computing. 2017;6(1):1–17. doi: 10.1093/comjnl/bxw050

[25] Tran DH, Tran NH, Pham C, Kazmi S, Huh EN, Hong CS. OaaS: offload as a service in fog networks. Computing. 2017;99(11):1081–1104. doi: 10.1007/s00607-017-0551-z

[26] Chang Z, Zhou Z, Ristaniemi T, Niu Z. Energy efficient optimization for computation offloading in fog computing system. In: GLOBECOM 2017-2017 IEEE Global Communications Conference. IEEE; 2017. p. 1–6.

[27] De Maio V, Brandic I. First hop mobile offloading of dag computations. In: 2018 18th IEEE/ACM International Symposium on Cluster, Cloud and Grid Computing (CCGRID). IEEE; 2018. p. 83–92.

[28] Hong ST, Kim H. QoE-aware computation offloading to capture energy-latency-pricing tradeoff in mobile clouds. IEEE Transactions on Mobile Computing. 2018;18(9):2174–2189. doi: 10.1109/TMC.2018.2871460

[29] Chen Y, Zhang N, Zhang Y, Chen X, Wu W, Shen X. Energy efficient dynamic offloading in mobile edge computing for internet of things. IEEE Transactions on Cloud Computing. 2019;9(3):1050–1060. doi: 10.1109/TCC.2019.2898657

[30] Liu Y, Su Z, Wang Y. Energy-Efficient and Physical Layer Secure Computation Offloading in Blockchain-Empowered Internet of Things. IEEE Internet of Things Journal. 2022;.

[31] Zhou H, Wu T, Chen X, He S, Guo D, Wu J. Reverse Auction-Based Computation Offloading and Resource Allocation in Mobile Cloud-Edge Computing. IEEE Transactions on Mobile Computing. 2022; p. 1–15. doi: 10.1109/TMC.2022.319349934970086

[32] Xu X, Li Y, Huang T, Xue Y, Peng K, Qi L, et al. An energy-aware computation offloading method for smart edge computing in wireless metropolitan area networks. Journal of Network and Computer Applications. 2019;133:75–85. doi: 10.1016/j.jnca.2019.02.008

[33] Hou X, Ren Z, Wang J, Cheng W, Ren Y, Chen KC, et al. Reliable computation offloading for edge-computing-enabled software-defined IoV. IEEE Internet of Things Journal. 2020;7(8):7097–7111. doi: 10.1109/JIOT.2020.2982292

[34] Dai S, Liwang M, Liu Y, Gao Z, Huang L, Du X. Hybrid quantum-behaved particle swarm optimization for mobile-edge computation offloading in internet of things. In: International Conference on Mobile Ad-Hoc and Sensor Networks. Springer; 2017. p. 350–364.

[35] Kuang L, Gong T, OuYang S, Gao H, Deng S. Offloading decision methods for multiple users with structured tasks in edge computing for smart cities. Future Generation Computer Systems. 2020;105:717–729. doi: 10.1016/j.future.2019.12.039

[36] Wang J, Wu W, Liao Z, Sherratt RS, Kim GJ, Alfarraj O, et al. A probability preferred priori offloading mechanism in mobile edge computing. IEEE Access. 2020;8:39758–39767. doi: 10.1109/ACCESS.2020.2975733

[37] Xu X, Zhang X, Gao H, Xue Y, Qi L, Dou W. BeCome: Blockchain-enabled computation offloading for IoT in mobile edge computing. IEEE Transactions on Industrial Informatics. 2019;16(6):4187–4195. doi: 10.1109/TII.2019.2936869

[38] Yang G, Hou L, He X, He D, Chan S, Guizani M. Offloading time optimization via Markov decision process in mobile-edge computing. IEEE Internet of Things Journal. 2020;8(4):2483–2493. doi: 10.1109/JIOT.2020.3033285

[39] Farahbakhsh F, Shahidinejad A, Ghobaei-Arani M. Multiuser context-aware computation offloading in mobile edge computing based on Bayesian learning automata. Transactions on Emerging Telecommunications Technologies. 2021;32(1):e4127. doi: 10.1002/ett.4127

[40] Huang L, Feng X, Feng A, Huang Y, Qian LP. Distributed deep learning-based offloading for mobile edge computing networks. Mobile Networks and Applications. 2018; p. 1–8.

[41] Wu S, Xia W, Cui W, Chao Q, Lan Z, Yan F, et al. An efficient offloading algorithm based on support vector machine for mobile edge computing in vehicular networks. In: 2018 10th International Conference on Wireless Communications and Signal Processing (WCSP). IEEE; 2018. p. 1–6.

[42] Sun W, Liu J, Yue Y. AI-enhanced offloading in edge computing: When machine learning meets industrial IoT. IEEE Network. 2019;33(5):68–74. doi: 10.1109/MNET.001.1800510

[43] Ali Z, Jiao L, Baker T, Abbas G, Abbas ZH, Khaf S. A deep learning approach for energy efficient computational offloading in mobile edge computing. IEEE Access. 2019;7:149623–149633. doi: 10.1109/ACCESS.2019.2947053

[44] Irshad A, Abbas ZH, Ali Z, Abbas G, Baker T, Al-Jumeily D. Wireless Powered Mobile Edge Computing Systems: Simultaneous Time Allocation and Offloading Policies. Electronics. 2021;10(8). doi: 10.3390/electronics10080965

[45] Shahidinejad A, Farahbakhsh F, Ghobaei-Arani M, Malik MH, Anwar T. Context-Aware Multi-User Offloading in Mobile Edge Computing: A Federated Learning-Based Approach. Journal of Grid Computing. 2021;19. doi: 10.1007/s10723-021-09559-x

[46] Ali Z, Abbas ZH, Abbas G, Numani A, Bilal M. Smart computational offloading for mobile edge computing in next-generation Internet of Things networks. Computer Networks. 2021;198:108356. doi: 10.1016/j.comnet.2021.108356

[47] Mao B, Tang F, Kawamoto Y, Kato N. Optimizing Computation Offloading in Satellite-UAV-Served 6G IoT: A Deep Learning Approach. IEEE Network. 2021;35(4):102–108. doi: 10.1109/MNET.011.2100097

[48] Xu J, Chen L, Ren S. Online Learning for Offloading and Autoscaling in Energy Harvesting Mobile Edge Computing. IEEE Transactions on Cognitive Communications and Networking. 2017;3(3):361–373. doi: 10.1109/TCCN.2017.2725277

[49] Dinh TQ, La QD, Quek TQS, Shin H. Learning for Computation Offloading in Mobile Edge Computing. IEEE Transactions on Communications. 2018;66(12):6353–6367. doi: 10.1109/TCOMM.2018.2866572

[50] Zhang K, Zhu Y, Leng S, He Y, Maharjan S, Zhang Y. Deep Learning Empowered Task Offloading for Mobile Edge Computing in Urban Informatics. IEEE Internet of Things Journal. 2019;6(5):7635–7647. doi: 10.1109/JIOT.2019.2903191

[51] Liu Y, Yu H, Xie S, Zhang Y. Deep Reinforcement Learning for Offloading and Resource Allocation in Vehicle Edge Computing and Networks. IEEE Transactions on Vehicular Technology. 2019;68(11):11158–11168. doi: 10.1109/TVT.2019.2935450

[52] Min M, Xiao L, Chen Y, Cheng P, Wu D, Zhuang W. Learning-Based Computation Offloading for IoT Devices With Energy Harvesting. IEEE Transactions on Vehicular Technology. 2019;68(2):1930–1941. doi: 10.1109/TVT.2018.2890685

[53] Huang L, Feng X, Zhang C, Qian L, Wu Y. Deep reinforcement learning-based joint task offloading and bandwidth allocation for multi-user mobile edge computing. Digital Communications and Networks. 2019;5(1):10–17. doi: 10.1016/j.dcan.2018.10.003

[54] Yu S, Chen X, Yang L, Wu D, Bennis M, Zhang J. Intelligent Edge: Leveraging Deep Imitation Learning for Mobile Edge Computation Offloading. IEEE Wireless Communications. 2020;27(1):92–99. doi: 10.1109/MWC.001.1900232

[55] Yu S, Chen X, Zhou Z, Gong X, Wu D. When Deep Reinforcement Learning Meets Federated Learning: Intelligent Multitimescale Resource Management for Multiaccess Edge Computing in 5G Ultradense Network. IEEE Internet of Things Journal. 2021;8(4):2238–2251. doi: 10.1109/JIOT.2020.3026589

[56] Liao L, Lai Y, Yang F, Zeng W. Online computation offloading with double reinforcement learning algorithm in mobile edge computing. Journal of Parallel and Distributed Computing. 2023;171:28–39. doi: 10.1016/j.jpdc.2022.09.006

[57] Mao B, Qiu J, Kato N. On an Intelligent Task Offloading Model to Jointly Optimize Latency and Energy for Electric Connected Vehicles. IEEE Transactions on Vehicular Technology. 2024;73(4):6024–6028. doi: 10.1109/TVT.2023.3333241

[58] Zhang Y, Yan L. Research on the optimization of energy consumption for multi-priority tasks in mobile computing offloading. Multimedia Tools and Applications. 2023;82(29):45453–45469. doi: 10.1007/s11042-023-15490-y

[59] Chen MH, Dong M, Liang B. Resource Sharing of a Computing Access Point for Multi-User Mobile Cloud Offloading with Delay Constraints. IEEE Transactions on Mobile Computing. 2018;17(12):2868–2881. doi: 10.1109/TMC.2018.2815533

[60] He X, Zhao K, Chu X. AutoML: A survey of the state-of-the-art. Knowledge-Based Systems. 2021;212:106622. doi: 10.1016/j.knosys.2020.106622

[61] Bano S, Hussain SF. Prediction of Covid-19 and post Covid-19 patients with reduced feature extraction using Machine Learning Techniques. In: 2021 International Conference on Frontiers of Information Technology (FIT); 2021. p. 37–42.

[62] Waqas M, Bano S, Hassan F, Tu S, Abbas G, Abbas ZH. Physical Layer Authentication Using Ensemble Learning Technique in Wireless Communications. Computers, Materials & Continua. 2022;73(3):4489–4499. doi: 10.32604/cmc.2022.029539

[63] Freund Y, Schapire RE. Experiments with a New Boosting Algorithm. In: In Proceedings of the Thirteenth International Conference on Machine Learning. Morgan Kaufmann; 1996. p. 148–156.

[64] Zhu Y, Mao B, Kato N. A Dynamic Task Scheduling Strategy for Multi-Access Edge Computing in IRS-Aided Vehicular Networks. IEEE Transactions on Emerging Topics in Computing. 2022;10(4):1761–1771. doi: 10.1109/TETC.2022.3153494

